# Study on dietary intake, risk assessment, and molecular toxicity mechanism of benzo[α]pyrene in college students in China Bashu area

**DOI:** 10.1002/fsn3.3007

**Published:** 2022-08-09

**Authors:** Yutong Ge, Hailian Yan, Xiaodong Shi, Zhixiang Wu, Yueteng Wang, Zelan Zhang, Qing Luo, Wei Liu, Li Liang, Lianxin Peng, Jianping Hu

**Affiliations:** ^1^ Key Laboratory of Coarse Cereal Processing, Ministry of Agriculture and Rural Affairs, School of Pharmacy, Sichuan Industrial Institute of Antibiotics Chengdu University Chengdu China

**Keywords:** benzo[α]pyrene, daily intake, molecular dynamics simulation, risk assessment, toxicity mechanism

## Abstract

As an extremely strong polycyclic aromatic hydrocarbon carcinogen, benzo[α]pyrene (BaP) is often produced during food processing at high temperatures. Recently, food safety, as well as toxicity mechanism and risk assessment of BaP, has received extensive attention. We first constructed the database of BaP pollution concentration in Chinese daily food with over 10^4^ data items; collected dietary intake data using online survey; then assessed dietary exposure risk; and finally revealed the possible toxicity mechanism through four comparative molecular dynamics (MD) simulations. The statistical results showed that the concentration of BaP in olive oil was the highest, followed by that in fried meat products. The margins of exposure and incremental lifetime cancer risk both indicated that the dietary exposure to BaP of the participants was generally safe, but there were still some people with certain carcinogenic risks. Specifically, the health risk of the core district population was higher than that of the noncore district in Bashu area, and the female postgraduate group was higher than the male group with bachelor degree or below. From MD trajectories, BaP binding does not affect the global motion of individual nucleic acid sequences, but local weak noncovalent interactions changed greatly; it also weakens molecular interactions of nucleic acid with *Bacillus stearothermophilus* DNA polymerase I large fragment (BF), and significantly changes the cavity structure of recognition interface. This work not only reveals the possible toxicity mechanism of BaP, but also provides theoretical guidance for the subsequent optimization of food safety standards and reference of rational diet.

## Graphical abstract

The margins of exposure (MOE) and incremental lifetime cancer risk (ILCR) both indicated that the dietary exposure to BaP of the participants was generally safe, but there were still some people with certain carcinogenic risks. From MD trajectories, BaP binding does not affect the global motion of individual nucleic acid sequences, but local weak non‐covalent interactions changed greatly; it also weakens molecular interactions of nucleic acid with Bacillus stearothermophilus DNA polymerase I large fragment (BF), and significantly changes the cavity structure of recognition interface.

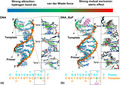

## 1 INTRODUCTION

According to *the Global Cancer Report* 2020 released by the World Health Organization (WHO) and the International Agency for Research on Cancer (IARC), there were 19.29 million new cases of cancer worldgh tumor epidemiological investigation and animal

wide, with 9.96 million deaths. In the case of China, the number of new infections and deaths each year is 4.57 and 8 million, the highest in the world. The high incidence of various gastrointestinal cancers, including gastric/colorectal/esophageal cancer, is closely related to unhealthy dietary patterns and food contamination (Feng et al., 2019). Benzo[α]pyrene (BaP) is one of the typical polycyclic aromatic hydrocarbon (PAH) compounds, which is known as the world’s three major carcinogens along with aflatoxin and nitrosamine. Humans are mainly exposed to BaP from breathing, skin contact, and food (Boström et al., 2002; Martorell et al., 2012), of which diet is the most important intake route (ACGIH, 2005). High‐temperature cooking is one of the main causes of the formation of PAHs (Purcaro et al., 2013). In terms of Chinese cooking methods (such as frying, grilling, etc.), BaP is easy to accumulate and becomes a potential factor for high health risks (Duan et al., 2016).

Traditionally, Bashu region is defined as the Sichuan Basin in southwest China and its surrounding areas, mainly containing Sichuan Province and Chongqing Municipality, as well as parts of Hubei, Hunan, Guizhou, Gansu, and other provinces, which is a densely populated and diet‐rich region; the core of Bashu region refers to Chengdu, the national central city food capital. Due to dietary habits, climate pollution, and other factors, Bashu area has become a high incidence area of gastric cancer and esophageal cancer (Yang et al., 2005). Deep‐fried and barbecued food—a local specialty—is popular among local residents including college students with advanced education. Frequent consumption of deep‐fried and barbecued food may not only lead to vomiting and diarrhea, but also has a positive correlation with the incidence of some metabolic diseases (such as hypertension, type 2 diabetes, obesity, etc.). In particular, excessive levels of trans‐fatty acids, acrylamide, and BaP are also associated with a greater risk of cancer (Ganesan et al., 2019).

Since a high incidence of scrotal cancer was reported among London chimney sweepers in 1775, the carcinogenicity of BaP has attracted increasing attention (Labianca, 1982). Through tumor epidemiological investigation and animal experiments, Alzohairy et al. (2021) and Li et al. (1996) found that BaP pollution was closely related to the occurrence of tumors, especially lung cancer, skin cancer, and colon cancer. According to the MTT (3‐(4,5‐dimethylthiazol‐2‐yl)‐2,5‐diphenyl‐2H‐tetrazolium bromide), Transwell, and scratch assays by Zhang et al. (2016), BaP significantly increased the migration and invasion of A549 cells and showed a significant correlation with lung cancer metastasis. Do and Byung (2017) differentiated human SH‐SY5Y neuroblastoma cells with all‐transretinoic acid (RA), while its growth was significantly inhibited after BaP addition. Experiments on mice conducted by the Qi group (Qi et al., 2020) showed that BaP exposure not only inhibited the function of neurotransmitter receptors and dopamine transporters, but also interfered with autophagy and increased the risk of Parkinson’s disease. Sheibani et al. (2020) evaluated the toxic effects of BaP on cell viability and steroid production through ovarian and brain cell culture, and the results showed that BaP altered the activity of aromatase (ARO) and disrupted the biosynthesis of estrogen. In terms of action mechanism, Hruszkewycz et al. (1992) incubated 7,8‐dihydrodiol 9,10‐epoxide of BaP and 16‐mer oligonucleotides together; the results show that BaP adducts inhibit the extension of the template chain, and the cis adduct is a more effective block to replication than a trans adduct. Alekseyev et al. (2001) investigated how BaP diol‐epoxide adducts block DNA replication in vitro through the gel‐retarding assay, and concluded that it may block conformational changes of ternary complexes, thus interfering with catalytic activity. Mg2+ is a critical cofactor working for the *Bacillus stearothermophilus* DNA polymerase I large fragment (BF) polymerase by chelating Asp860 and Asp653 amino acids to stabilize the catalytic domain, which helps to locate the reactive material as deoxynucleoside triphosphate (dNTP) (Johnson et al., 2003). According to a series of structural biology and molecular simulation studies on BaP‐deoxyguanosate (BaP‐dG) adducts and their interactions with DNA polymerase I, the adducts induce the disruption of DNA replication (Alekseyev et al., 2001; Lipinski et al., 1998; Wu et al., 2020).

In the field of content determination, Ndadani (2016) evaluated the BaP contamination of sardine, mackerel, anchovy, and horse mackerel species that originate from the Atlantic and Mediterranean Oceans with gas chromatography coupled with mass spectrometry (GC–MS); the results met the global essential safety requirements of the European Commission. Nie et al. (2014) conducted a questionnaire survey on the diet of people in Taiyuan China, and assessed the cancer risk of 16 kinds of PAHs including BaP; the results show that the content of PAHs increases significantly during heating and frying process, and the risk of cooked food is much higher than that of raw food. Zhu et al. (2019) statistically analyzed the dietary exposure of PAHs including BaP in southwest China, and then conducted a risk assessment based on incremental lifetime cancer risk (ILCR) parameters, finally finding that BaP was a potential exposure source of high cancer risk in this area. The margins of exposure (MOE) model was used to assess the health risks of BaP in edible vegetable oils, which showed a high carcinogenic potential (Niu et al., 2021). Cao et al. (2021) and Zhou et al. (2018) collected soil samples from the central/eastern Qinghai‐Tibetan Plateau, as well as the Chaohu watershed located in the lower reaches of the Yangtze River; the concentration characteristics and sources of PAHs including BaP were measured with GC–MS, suggesting that a small portion of the soils in the two areas have potential ecological risk. In terms of anti‐BaP toxicity, Kolade and Oladiji (2018) found that the biochemical indexes of BaP‐induced albino rats were significantly reversed after curcumin administration. BaP can inhibit the osteogenic differentiation of adipogenic stem cells (Labianca, 1982); Park et al. (2021) from South Korea found that the extract from black jade could counteract this toxic process through the anti‐apoptotic mechanism by quantitative polymerase chain reaction (qPCR) and cell experiments. Yuan et al. (2021) identified an omega glutathione S‐transferase gene encoding 256 amino acids from Polychaete Aibuhitensis, which plays an important role in the degradation of BaP. According to GB 2762‐2017, the limit of BaP for grain and its products, meat and meat products, aquatic animals and their products is 5.0 μg kg^−1^; that for oil and its products is <10.0 μg kg^−1^, higher than the European Union (EU) standard of 2.0 μg kg^−1^ for edible vegetable oil.

Although there have been many previous studies on BaP cytotoxicity, action mechanism, concentration determination, detoxification strategies, and standard establishment, systematic studies incorporating BaP concentration databases, dietary exposure, risk assessment, and toxicity mechanisms have not been reported. In this article, we first established a BaP concentration database, collected the daily dietary intake of college students in Bashu area by online survey, and then analyzed dietary risk by combining point evaluation and probabilistic assessment strategies. Finally, the possible toxicity mechanism of BaP was proposed based on the comparative analyses of weak interactions and binding free energy calculated from molecular dyanamics (MD) trajectories. This work aims at providing theoretical guidance for the subsequent establishment of oil‐processing food safety standards and health‐based dietary intake habits.

## 2 MATERIALS AND METHODS

### 2.1 Data collection and questionnaire survey

The MySQL database of BaP concentration in different foods in China was constructed based on all the available experimental literature from 1986 to 2020. The database has possessed 10 common functions such as information input, data import, data export, data modification, browsing, query, sorting, general screening, conditional screening, and printing. The contents of the form include seven parts: food type, hazardous substance type, detection site, detection time, BaP concentration, national limit standard, and data sources.

The bookkeeping method was used for the food‐intake questionnaire survey. The dietary intake of 637 college students from the week before the survey (including Saturday and Sunday, a total of 7 days) was collected. The participants were asked to report their gender, body weight, height, age, education, dietary places as well as dietary frequency.

### 2.2 Risk assessment models

Based on the concentration database and food‐intake questionnaire data, the dietary exposure risk of BaP was analyzed using both point evaluation and probabilistic assessment models. Health risk can be assessed by exposure daily intake (*EDI*), calculated as food consumption (*IR*, kg day^−1^) multiplied by residual concentration of risk substance in food (*C*, mg kg^−1^) divided by body weight (*BW*, kg), as shown in Equation 1. As a typical health‐risk assessment method, *EDI* of PAH BaP was calculated to describe the harm degree to human body according to the Exposure Factors Handbook (USEPA, 1997).

(1) $$\mathrm{EDI}=\frac{C\times \mathrm{IR}}{\mathrm{BW}}$$

In accordance with the recommendations of the European Food Safety Authority (EFSA) for a unified approach to the assessment of genotoxic and carcinogenic substances (EFSA, 2005), two representative methods were adopted to calculate the MOEs comparing the animal potency data with human exposure scenarios.

(2) $${\mathrm{MOE}}_{T25}=\frac{T25}{\mathrm{EDI}}$$

(3) $${\mathrm{MOE}}_{\text{BMDL}10}=\frac{\text{BMDL}10}{\mathrm{EDI}}$$

As can be seen from Equations 2 and 3, two reference points on the dose–response relationship are referenced for the MOE calculation: (1) the T25 value, set as 2.4 × 10^6^ ng kg^−1^ day^−1^, represents a chronic daily dose that causes tumors at specific tissue sites in more than 25% of the experimental animals, as determined by linear extrapolation of the lowest dose (Dybing et al., 1997; O'Brien et al., 2006); (2) the BMDL10, defined as a 95% confidence interval for a baseline dose that caused a 10% increase in tumor incidence of laboratory animals, is derived from mathematical modeling of the dose–response data, with a default value of 7 × 10^5^ ng kg^−1^ day^−1^ (Barlow et al., 2006; Martinez‐Miranda et al., 2019).

The above MOE_T25_ and MOE_BMDL10_ both are point evaluation models with the advantages of speed, but they also rely too much on the accuracy of parameters, failing to provide the possible range and random factors of exposure, thus they are mainly used for data screening and conservative estimation. In the analysis of a specific ILCR, Monte Carlo (MC) sampling in Crystal Ball package was used to process the sampled data to ensure a more accurate dietary assessment. The calculation of ILCR is shown as:

(4) $$\text{ILCR}=\frac{C\times \mathrm{IR}\times \mathrm{EF}\times \mathrm{ED}\times {\mathrm{CSF}}_{\mathrm{BaP}}}{\mathrm{BW}\times \mathrm{AT}}=\frac{\mathrm{Ed}\times \mathrm{EF}\times \mathrm{ED}\times {\mathrm{CSF}}_{\mathrm{BaP}}}{\mathrm{BW}\times \mathrm{AT}}$$

where *C* and *IR* are the residual concentrations of BaP in food in mg kg^−1^ and food consumption in kg day^−1^, respectively. The parameter *Ed* is the diary intake of BaP in mg day^−1^, obtained by multiplying *C* and *IR*; parameters EF, ED, and BW represent the exposure frequency with default value of 365 days year^−1^, adult exposure duration with default value of 43 years, and body weight in kg, respectively; *CSF*_BaP_ is the oral cancer slope factor of BaP (7.3 kg day mg^−1^) and *AT* is used to describe the average lifespan for carcinogens (25,550 days) (Conti et al., 2012; Xia et al., 2010; Yu et al., 2015; Zhu et al., 2019). In particular, ED and BW both show logarithmic normal (lognormal) distribution. The probability distribution range of the ILCR can be obtained through 10^4^ iterations of MC simulations.

### 2.3 Molecular dynamics simulations

The thermophilic *Bacillus stearothermophilus* DNA polymerase I large fragment (BF) complexes with normal and BaP‐bound DNA duplexes (PDB IDs: 1L5U and 1XC9) were selected from the protein data bank (PDB) (Hsu et al., 2005; Johnson et al., 2003); both protein sequences are the same, Ala297‐Lys876, while the length and primary structure of two nucleic acid sequences are different. In order to make a better comparison, the nucleic acid system, namely ^P^G1‐^T^G22, was determined uniformly according to the length of 1XC9 nucleic acid and the primary sequence of 1L5U nucleic acid. In this work, four comparative MD simulations were performed for the BF_DNA_BaP, BF_DNA, DNA_BaP, and DNA systems for 500 ns at 300 K using AMBER 18 package (Case et al., 2018). Here, the BF_DNA_BaP system includes BF (Ala297‐Lys876), nucleic acid sequence (^P^G1‐^T^G22), and BaP, where BaP is covalently chelated with ^T^G12; BF_DNA and DNA_BaP were obtained by removing BaP or BF based on BF_DNA_BaP, respectively. Systems that contain only nucleic acid sequences are represented by DNA. The simulation temperature was set at 300 K and the TIP3P (transferable intermolecular potential with 3 points) water model (Jorgensen et al., 1983) was applied. Total 3521/4787/16094/16013 water molecules and 20/20/32/32 Na^+^ counterions were added to the DNA, DNA_BaP, BF_DNA, and BF_DNA_BaP systems, respectively, with the box boundary of 10 Å (Wu et al., 2020).

The four systems were optimized twice subsequently. The first is to constrain the solute (force constant is 2.09 × 10^5^ kJ mol^−1^ nm^−2^) and optimize the solvent, consisting of a 5000‐step steepest descent and a 5000‐step conjugate gradient procedure; then in case of complete removal of the constraints, another 5000‐step steepest descent and 5000‐step conjugate gradient optimization were performed. The energy convergence threshold of the two minimizations was <4.182 × 10^−4^ kJ mol^−1^ nm^−2^. After the energy minimization was completed, the productive MD simulations were started, which were also divided into two procedures. First of all, the solute was constrained with the force constant of 41.82 kJ mol^−1^ nm^−2^ and the temperature gradually increased from 0 to 300 K in the initial 5 ns. Afterwards, a 95 ns unconstrained MD simulation was performed adopting SHAKE algorithm (Ryckaert et al., 1977) to constrain the hydrogen‐containing atoms with nonbonded interaction radius of 10 Å. The integration step was set as 2 fs and the conformation was collected every 10 ps, thus total 5 × 10^5^ conformations were collected for the following structural analyses.

### 2.4 Free energy landscape

The main idea of free energy landscape (FEL) (Liwo et al., 1997) is to investigate molecular motion and conformational changes for a biomacromolecular system, by comparing the minimum value of free energy surface (corresponding to the most stable state of the system) and demarcation point (related to a transient state in the process of conformational transition). As a common dimension reduction algorithm in data mining, principal component analysis is widely used to describe the most important global functional motion of proteins and nucleic acids. The first principal component (PC1) and the second principal component (PC2) both serve as reaction coordinates for the mapping of free energy surface diagram (Hegger et al., 2007). Free energy is defined as:

(5) $$∆G\left(X\right)=-K_{B}T\ln P\left(X\right)$$

Here, the reaction coordinate *X* is PC1, 2 and *k*_*B*_ is the Boltzmann constant. T expresses the absolute temperature in Kelvin, and *P(X)* is the probability of conformational distribution representing the contribution of a particular conformation to the overall PCs. In this work, conformational information extraction of PC1 and PC2 was the basis for FEL analysis to investigate conformational change of DNA and DNA_BaP systems.

### 2.5 Weak noncovalent interactions

The interactions in chemical systems are mainly divided into chemical bonds and noncovalent bonds. Chemical bonds consist of covalent bonds and ionic bonds, which are defined as strong interactions with a relatively high bond strength; the latter is generally weaker, usually an order of magnitude weaker than the former. Hydrogen bond/halogen bond/pi–pi stack/van der Waals forces/steric hindrance all belong to medium‐ and long‐range interactions from distance, as well as weak interactions from the energy perspective. For chemical small molecule systems, the expensive MP2/6–311 + G^**^ is often used to calculate the reduced density gradient (RDG) function isosurface to represent the weak interaction region (Johnson et al., 2010). The equation to calculate RDG is as follows:

(6) $$\mathrm{RDG}=\frac{1}{2\times {\left(3\times \pi^{2}\right)}^{1/3}}\times \frac{|\nabla \rho \left(r\right)|}{\rho {\left(r\right)}^{4/3}}$$

where ∇ is the gradient operator and $|\nabla \rho \left(r\right)|$ is the norm of the electron density gradient. Generally, RDG decreases first and then increases gradually from the vicinity of nucleus to the chemical bond (with the minimum RDG value), weak interaction region, and molecular edge. Compared with the bonding region, the difference between the actual‐electron density and the free‐electron one in the weak noncovalent interaction region is relatively small. To biological macromolecular systems such as proteins and nucleic acids, the free‐electron density is often determined as a real‐space function, and the weak interaction regions can be qualitatively characterized by quickly calculating their approximate density sum (Gironés et al., 2001). Based on the RDG theory and free‐electron density approximation, the changes of weak noncovalent interactions for double‐stranded nucleic acid sequences were compared after the association with BaP via Multiwfn package (Lu & Chen, 2012).

### 2.6 Prediction of binding free energy

The conformations were extracted from the two MD equilibrium trajectories of the BF_DNA and BF_DNA_BaP systems at 0.05 ns intervals from 50 to 100 ns. Based on the 1000 snapshots, the average binding free energy between BF and its nucleic acid substrate was calculated with Molecular Mechanics/Poisson Boltzmann Surface Area (MM/PBSA) method (Sun et al., 2019). The formula is as follows:

(7) $$∆G_{\text{bind}}=∆H-T∆S=\left(∆E_{\mathrm{VDW}}+∆E_{\mathrm{ELE}}+∆G_{\text{PBELE}}+∆G_{\text{PBSUR}}\right)-T∆S$$

where Δ*E*_*VDW*_ refers to the nonpolar fraction of intramolecular energy under vacuum, while Δ*E*_ELE_ indicates the electrostatic section; Δ*G*_PBELE_ and Δ*G*_PBSUR_ correspond to the hydrophilic and hydrophobic parts of the solvation binding free energy, respectively; Δ*H* represents the total enthalpy change and *T* is the absolute temperature in Kelvin; Δ*S* refers to the total entropy change calculated using the normal mode method. In addition, energy decomposition was performed to divide the binding energy into various residues, and the key residues in BF system identifying the nucleic acid substrate can be obtained.

## 3 RESULTS AND DISCUSSION

### 3.1 Evaluation of BaP in different food samples

By querying China food safety big data platform (http://food.chinaii.cn/), China agricultural and rural big data (http://www.agdata.cn/), China food safety database (http://www.loodns.com/zz11952.html), Food partner network food database (http://db.foodmate.net/), China general administration of customs (http://www.customs.gov.cn), National bureau of statistics of China (http://www.stats.gov.cn/), and Ministry of agriculture and rural affairs of China (http://www.moa.gov.cn/), and referring to all available literature on BaP concentration data in China from 1986 to 2020, the MySQL database of BaP pollution concentration in Chinese processed food (food_hazardous_substance_sql.xlsx in Appendix S1) was established.

Table S1 lists 17 kinds of daily food with a high BaP concentration, mainly distributed in provinces Sichuan, Chongqing, Hubei, Hunan, Guizhou, Gansu, Fujian, Ningxia, Guangdong, Guangxi, Nei Mongol, Shaanxi, Henan, Shanxi, Heilongjiang, and Jilin in China; The limit standard in Table S1 refers to the GB 2762‐2017 safety threshold for this type of food; it is worth mentioning that all of these data were determined by high performance liquid chromatography (HPLC) with fluorescence detection (Cao et al., 2009; Cheng et al., 2015; Hao, 2015; Hu et al., 2018; Huang et al., 2018; Jiang & Wang, 2017; Liu & Wang, 2020; Luo et al., 2015; Shi et al., 2012; Wang et al., 2020; Wu et al., 2012; Yang et al., 2019; Zhang et al., 2010; Zhao et al., 2017). Obviously, from the point of view of data source, it is basically consistent with Bashu area of China; thus, to carry out BaP dietary exposure and risk assessment for the most creative college students in this area has a good basis and certain guiding significance for healthy diet.

**FIGURE 1:**
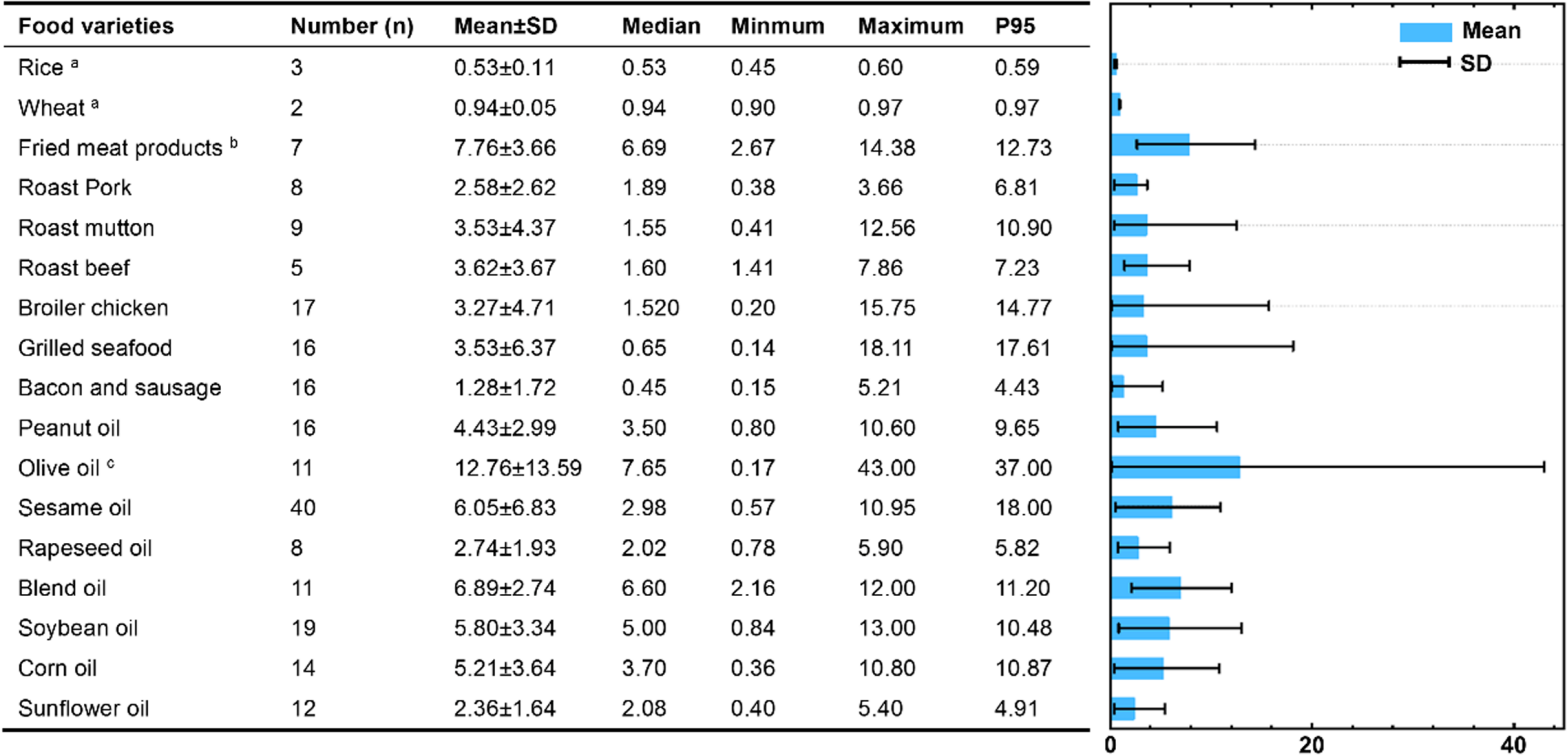
Benzo[α]pyrene (BaP) concentrations in various daily foods (μg kg^−1^). The comparison between the three data groups a, b, and c is statistically significant (*p* < .05).

According to the BaP concentration of 17 main foods listed in Table S1, their statistical data are presented in Figure 1. The concentration of BaP in food ranged from 0.14 to 43.00 μg kg^−1^, and olive oil was the highest with an average of 12.755 μg kg^−1^. Interestingly, the maximum and minimum values of BaP concentration detected in olive oil were 43.00 and 0.17 μg kg^−1^, showing the largest standard deviation. In addition, the BaP concentration in fried meat products was only lower than that in olive oil, with an average value of 7.76 μg kg^−1^; sure enough, that in rice and wheat without oil processing was lower, averaging 0.53 and 0.94 μg kg^−1^, respectively. After the t‐test, the comparison between the three data groups mentioned above, namely rice/wheat, fried meat products, and olive oil, is statistically significant (*p* < .05).

### 3.2 Demographic analysis of questionnaire participants

**FIGURE 2:**
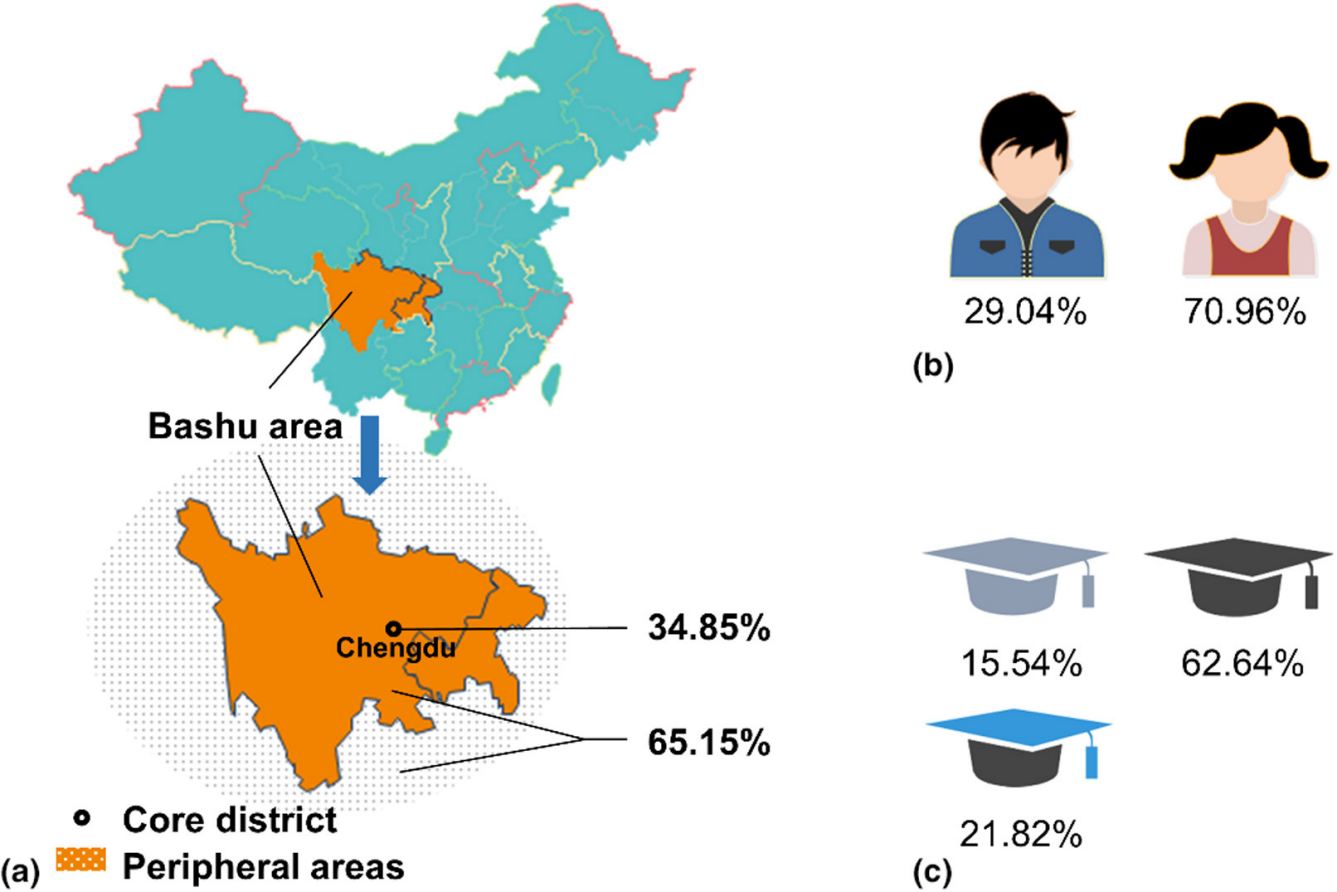
The location, gender, and educational qualification of the questionnaire participants.

A questionnaire (https://www.wjx.cn/wxloj/datafullscreen.aspx?activity=94141897, see the additional file questionnaire.xlsx in Appendix S2) was used to assist in determining the dietary habits and daily intake of BaP for college students in China Bashu area. After collection of the questionnaire, participants weighed between 38 and 90 kg with an average of 63.46 kg; their height ranged from 148 to 195 cm with an average of 158.50 cm; age ranged from 15 to 33 years old with a mean of 19.4. Figure 2 shows the location, gender, and education of the questionnaire participants. As seen from Figure 2a, there are 222 participants in the core district (i.e., Chengdu, Sichuan province) of Bashu region, accounting for 34.85%; the remaining 415 people (about 65.15%) are distributed in the noncore peripheral areas (i.e., the surrounding areas of Chengdu). According to Figure 2b,c, the number of male/female participants was 185/452, accounting for 29.04%/70.96%, respectively; moreover, the number of junior college/bachelor/postgraduate students was 99/399/139, accounting for 15.54%/62.64%/21.82%, respectively.

### 3.3 Dietary exposure to BaP and its risk assessment

The risk of dietary exposure to BaP was analyzed by combining the point evaluation and nonparametric probability assessment strategies, i.e., the MOE and ILCR for different groups of participants both were calculated and compared. According to the BaP concentration provided in the established database and dietary intake in the questionnaire, three key parameters (i.e., EDI, MOE_T25_, and MOE_BMDL10_) of participant groups were calculated using formula (1), (2), (3).

Table 1 lists the risk assessment of BaP performed with MOE_T25_ and MOE_BMDL10_ point assessment models. As shown from Table 1, the average MOE_BMDL10_ of the participants was 33,549, exceeding the low‐risk MOE reference value of 10^4^ recommended by the European Food Safety Authority (EFSA), indicating the overall diet safety. By comparing the regional distribution of MOE, the dietary exposure in the core district of Bashu is relatively higher than that in the noncore area, which is related to the high frequency of fried meat consumption according to the questionnaire survey. In terms of gender, the MOE value of females was relatively lower, and the BaP dietary exposure was slightly higher; despite having a more varied diet than males, females were more likely to eat fried and grilled foods. Most of the participants with college degree ate in the canteen; those with bachelor degree or above ate in more places, such as restaurants and outdoor vendors; this partly explains why high‐educated people have a higher risk of dietary exposure to BaP. It is worth mentioning that there are some differences between mean and median for both EDI and MOE data, which are related to the small sample size.

**TABLE 1:** The risk assessment of benzo[α]pyrene (BaP) performed by MOE_T25_ and MOE_BMDL10_ point assessment models

Population group	Number (*n*)	EDI (ng kg^−1^ day^−1^)			MOE_T25_			MOE_BMDL10_		
		Mean	Median	P95	Mean	Median	P95	Mean	Median	P95
All	637	7.10	5.20	20.63	1,150,259	457,660	4,467,387	33,549	13,348	130,299
Core district	222	7.75	5.62	20.92	931,294	426,788	3,255,343	27,163	12,448	94,947
Peripheral areas	415	6.75	4.75	20.55	1,270,284	486,152	4,707,937	37,050	14,179	137,315
Male	185	6.41	4.09	17.99	1,428,217	566,701	6,802,933	41,656	16,529	198,419
Female	452	7.38	5.43	20.76	1,038,330	436,318	3,520,169	30,285	12,726	102,672
Junior college	99	5.85	4.36	15.81	1,223,927	549,234	5,160,697	35,698	16,019	150,520
Bachelor	399	7.05	5.07	20.66	1,183,753	460,620	4,238,164	34,526	13,435	123,613
Postgraduate	139	8.13	6.02	21.21	1,003,045	397,267	3,097,819	29,255	11,587	90,353

**FIGURE 3:**
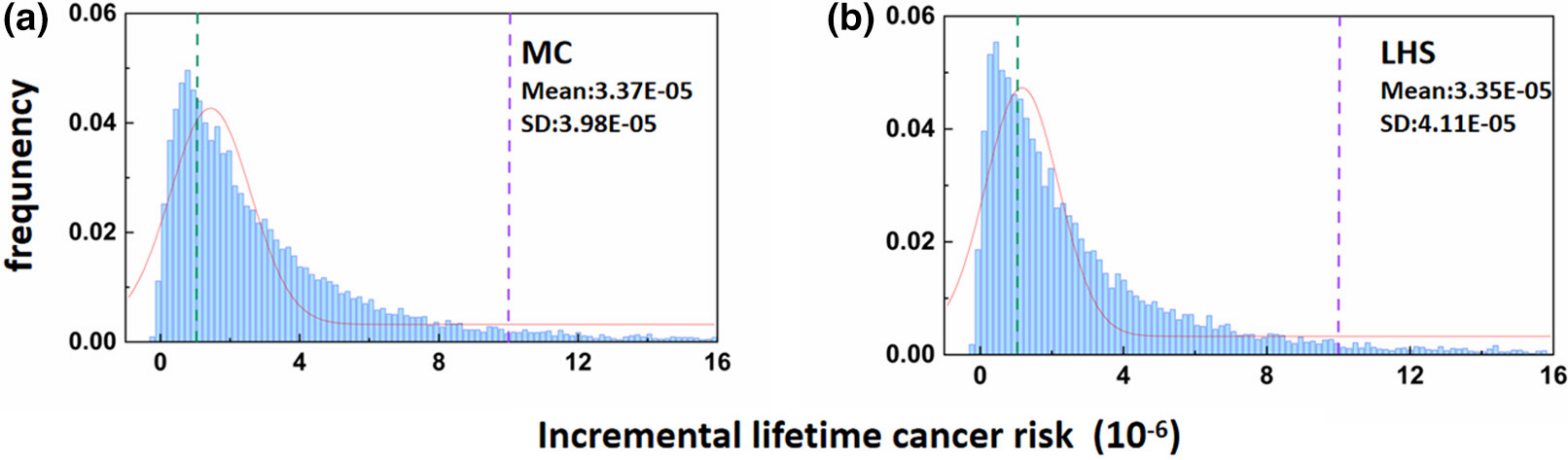
Distribution of incremental lifetime cancer risk (ILCR) for college students in China Bashu area using Monte Carlo (MC) (a) simulation and Latin hypercube sampling (LHS) (b). Green and purple vertical lines represent the U.S. Environmental Protection Agency (USEPA) acceptable risk level and serious risk level, respectively. Red curve represents the logarithmic normal (lognormal) distribution.

In order to better characterize cancer risk specifically, nonparametric probability assessment ILCR value was predicted using Equation 4. According to the U.S. Environmental Agency (USEPA), ILCR of 10^−6^ is the threshold for acceptable cancer risk and that greater than 10^−4^ indicates a serious risk; the lower the ILCR value, the lower the risk of cancer. Figure 3 shows the ILCR distribution of BaP exposure for college students in China Bashu area using Monte Carlo (MC) simulation and Latin hypercube sampling (LHS). As can be seen from Figure 3, the average ILCRs of the population calculated by the two methods are 3.37 × 10^−5^ and 3.35 × 10^−5^, respectively; the data are self‐consistent and reliable; considering that the value is between 10^−6^ and 10^−4^, indicating that there is a certain carcinogenic risk associated with the daily intake of BaP.

**FIGURE 4:**
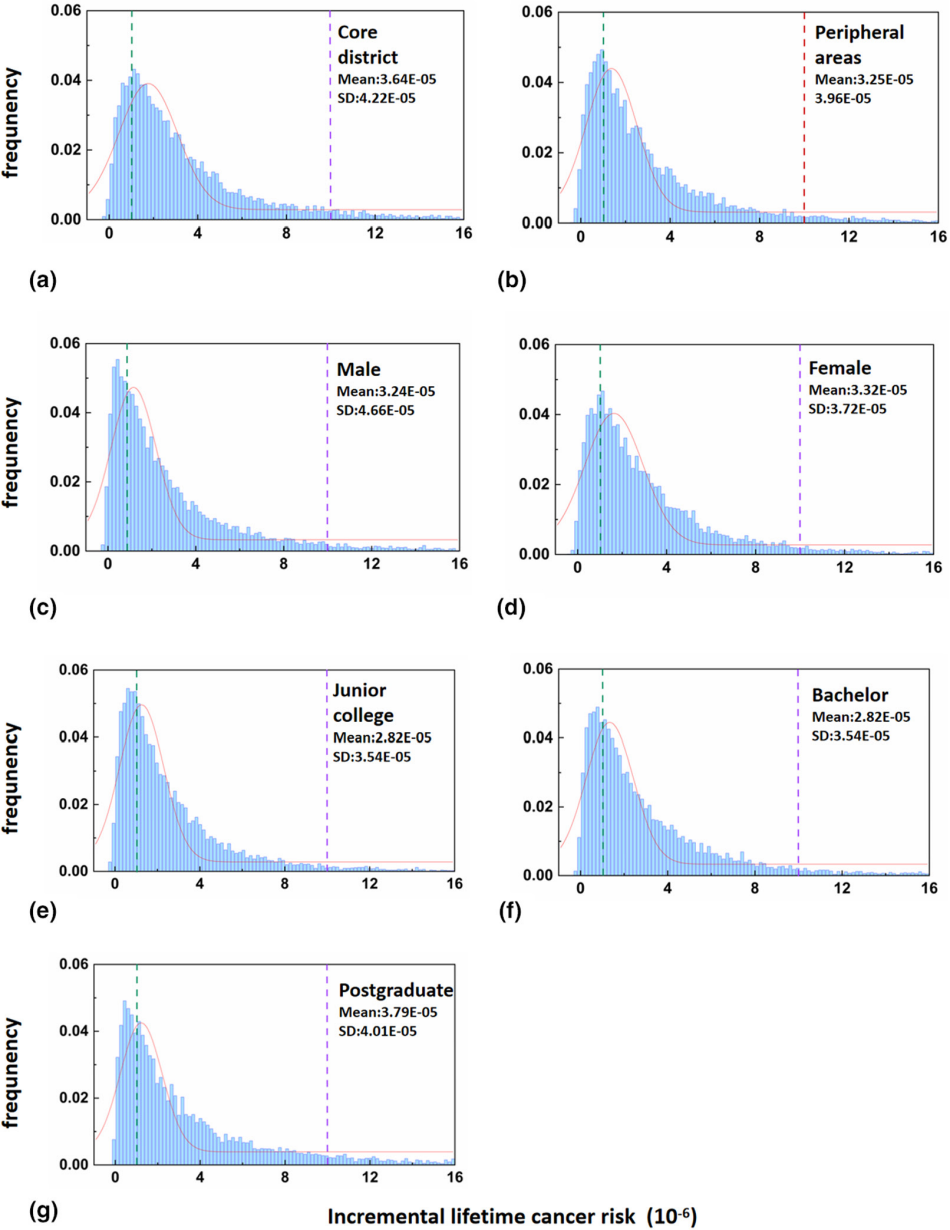
Distributions of incremental lifetime cancer risk (ILCR) for the three population groups derived using Monte Carlo (MC) simulation: (a,b) core district vs. peripheral area; (c,d) male vs. female; and (e–g) college, bachelor vs. postgraduate. The meaning of line colors is the same as illustrated in Figure 3.

As shown in Figure 4a,b, the average ILCR of the population in core district and noncore areas is 3.64 × 10^−5^ and 3.25 × 10^−5^, respectively, indicating that the cancer risk of the population in core area is higher than that in noncore area; Low‐risk, medium‐risk, and high‐risk groups in core district accounted for 18.82%, 74.30%, and 6.33%, while those in noncore areas accounted for 24.70%, 68.12%, and 5.52%. As shown in Figure 4c,d, the average ILCRs of male and female are 3.24 × 10^−5^ and 3.32 × 10^−5^, respectively, indicating that the cancer risk of female in the survey population is higher than that of male. Interestingly, the proportion of males at low‐risk, medium‐risk, and high‐risk was 28.50%, 64.11%, and 5.60%, while that of females was 21.43%, 72.85%, and 4.84%, which shows that there was an even larger percentage of males at high‐risk. In Figure 4e–g, the mean ILCRs of participants with college, bachelor, and graduate degrees were 2.82 × 10^−5^, 3.30 × 10^−5^, and 3.79 × 10^−5^, respectively. The proportions at low‐risk, medium‐risk, and high‐risk for the three groups of educational background were 28.14%/67.01%/3.63%, 25.49%/68.49%/5.08%, and 24.77%/63.94%/10.82%. It suggests that the cancer risk was the highest for those with a graduate degree, while lower for those with college degrees.

### 3.4 Possible toxicity mechanism of BaP

#### 3.4.1 Altering the conventional weak noncovalent interactions of double‐stranded nucleic acid

**FIGURE 5:**
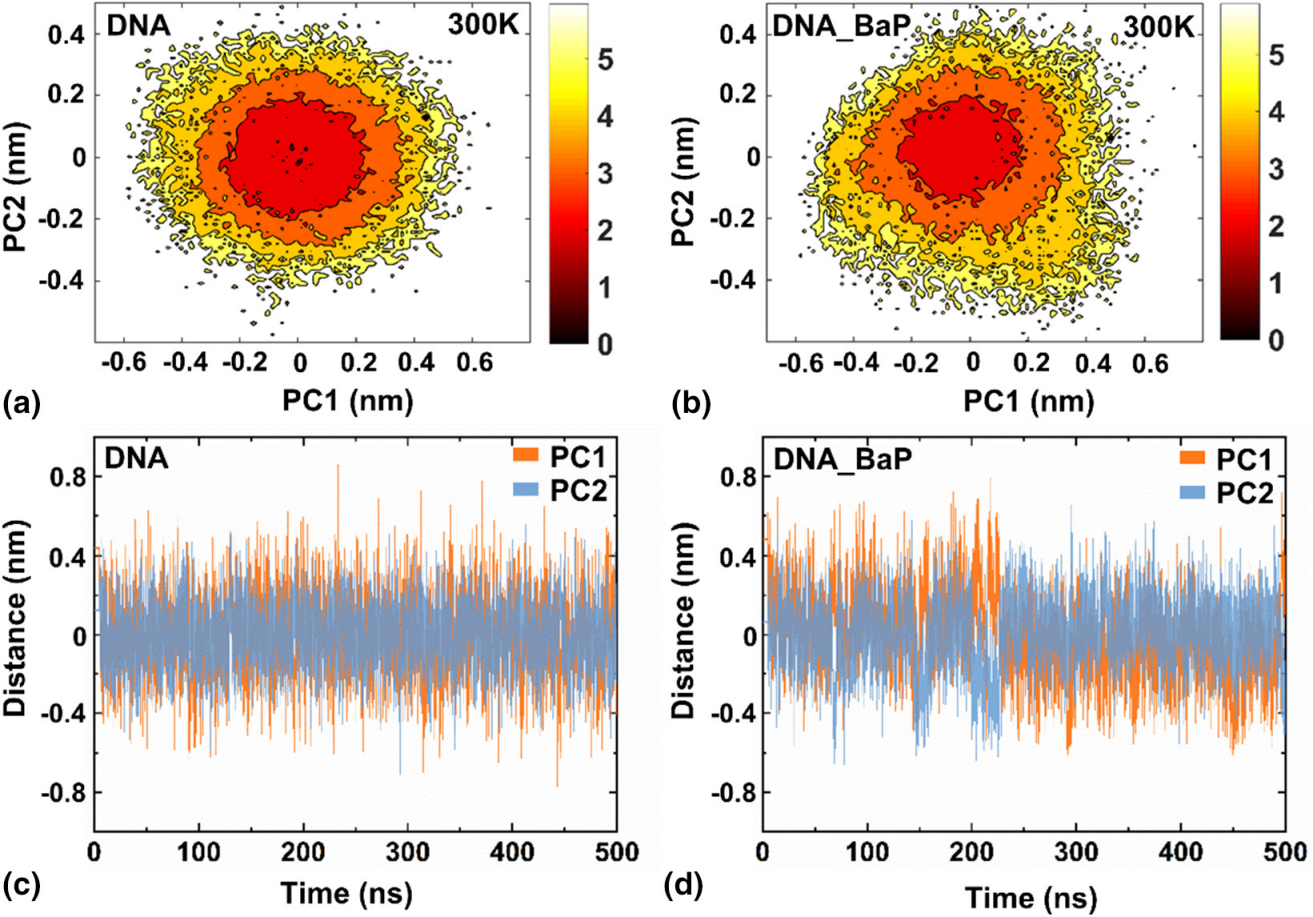
Free energy landscapes and conformational fluctuation of the top two principal components for the DNA (a,c) and DNA_BaP (b,d) systems.

Figure 5a and b, respectively, show the distribution of conformational free energy for the DNA and DNA_BAP systems at 300 K, where the darker the color, the lower the free energy. It can be seen that the fluctuation range of PC1 and PC2 of DNA is −0.6 ~ 0.6/ ‐0.4 ~ 0.4 nm, showing a concentric circle‐like distribution, which is consistent with previous reports that double‐stranded nucleic acid sequences are dominated by simple spiral motion (Hu & Wang, 2010); the same movement pattern was observed in the DNA_BaP system. As shown from Figure 5a,b, there is an independent region with lower free energy in each of the two systems, corresponding to the dark red circle. Figure 5c,d shows conformational fluctuation of the top two principal components for the DNA and DNA_BaP systems. Its range was between −0.2 and 0.2 nm consistent with the radius of the dark red circle in Figure 5a,b, showing a typical Gaussian distribution without obvious functional motion characteristics. By comparing the motion range between DNA and DNA_BaP systems, it can be inferred that the association of BaP does not affect conformational sampling space and global motion pattern of single double‐stranded nucleic acid sequences.

**FIGURE 6:**
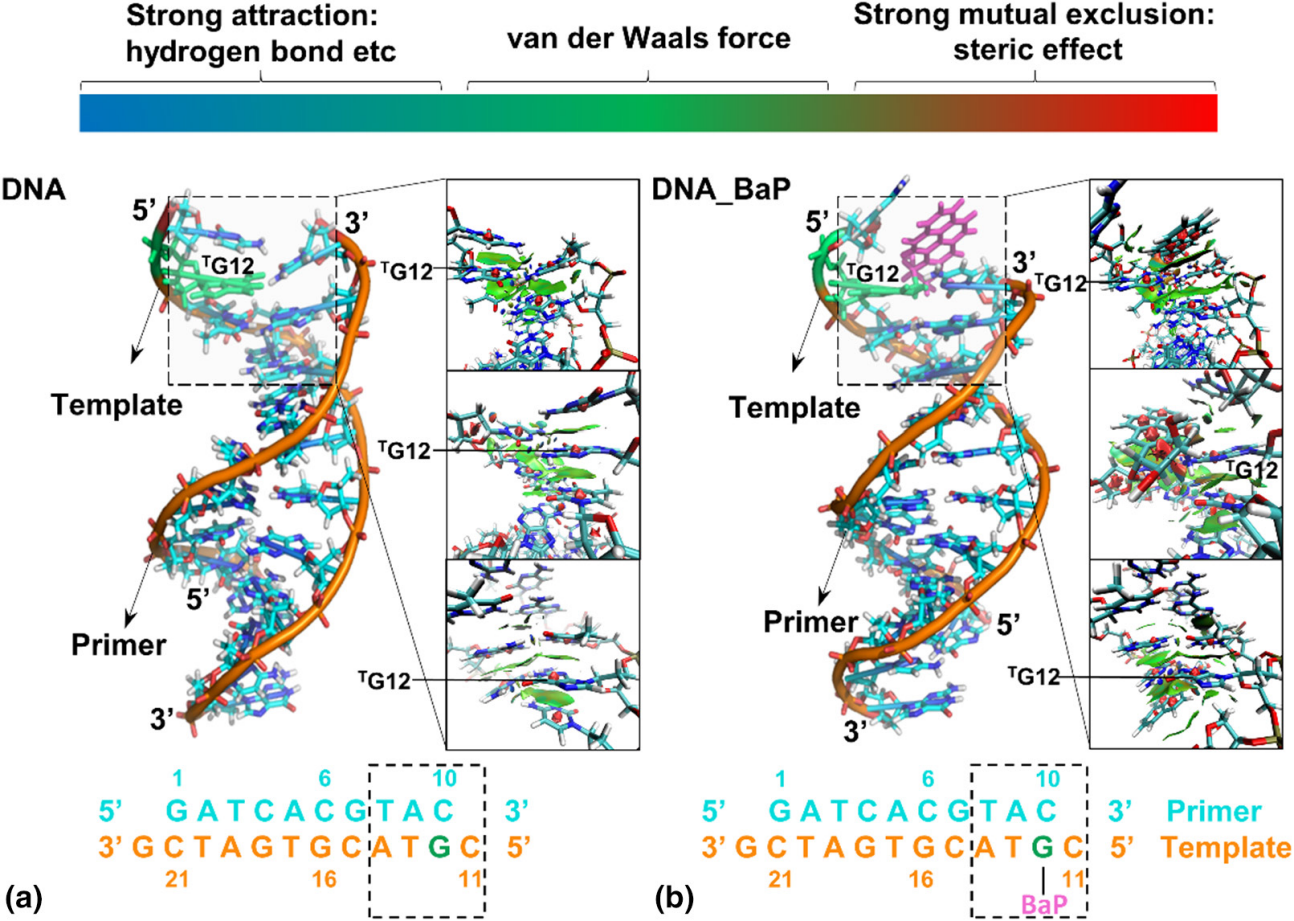
The weak noncovalent interactions of the DNA (a) and DNA_BaP (b) systems.

Although BaP binding does not affect the overall motion pattern of individual nucleic acid sequences, what does it do to their local conformation? Figure 6 shows the weak noncovalent interactions of the DNA and DNA_BaP systems; Blue, green, and red are, respectively, used to describe the strong attraction including hydrogen bonds, van der Waals force, and a strong mutual exclusion including steric effect; the larger the color area, the stronger the force. As shown from Figure 6, after covalently binding BaP, the van der Waals interactions of ^T^G12‐^P^C10 with its adjacent bases (^T^C11) and base pairs (^T^T13‐^P^A9) became stronger, and a strong hydrogen bond attraction effect and a strong steric hindrance mutual repulsion were both introduced. In addition, the van der Waals forces between ^T^G12 and ^P^C10, as well as between ^T^T13 and ^P^A9, disappeared and were replaced by a series of strong mutual exclusions. In a word, the covalent binding of BaP significantly affects the weak noncovalent interactions of nucleic acid sequence, which is particularly obvious between the upper and lower base pairs near the binding site, as well as between the left and right bases. It is consistent with our previously reported increase in torsion angle fluctuation at BaP chelation (Wu et al., 2020).

#### 3.4.2 Weakening molecular recognition between Polymerase I and double‐stranded nucleic acid

In order to further explore how BaP inhibits the biological functions of Polymerase I, Table 2 lists the calculated binding free energies and their compositions. The definitions of *E*_ELE_, *E*_VDW_, *G*_PBSUR_, and *G*_PBCA_ in Table 2 are shown in Equation 7. Compared with the BF_DNA system, the binding free energy between protein and nucleic acid sequences in the BF_DNA_BAP system increased from −87.56 to −33.61 kcal mol^−1^, indicating that the association of BaP significantly weakened molecular recognition between BF and DNA (especially the electrostatic interaction, *E*_ELE_ + *G*_PBCAL_), and then reduced the catalytic efficiency of BF. After all, maintaining stable interactions with DNA is essential for Polymerase I to perform its biological functions.

**TABLE 2:** Calculated binding free energies and their compositions in BF_DNA and BF_DNA_BaP (kcal Mol^−1^)

Items	BF_DNA		BF_DNA_BaP	
	Mean	Std.	Mean	Std.
*E*_ELE_	−605.07	113.75	2.21	45.18
*E*_VDW_	−195.04	9.82	−192.72	9.64
*G*_PBSUR_	−21.74	0.55	−22.65	0.47
*G*_PBCAL_	662.78	112.83	114.46	49.99
Δ*H*	−159.06	5.99	−98.70	3.22
*T*Δ*S*	−71.51	5.79	−65.10	8.05
${∆G}_{\text{bind}}^{\mathrm{cal}}$	−87.56	–	−33.61	–

**FIGURE 7:**
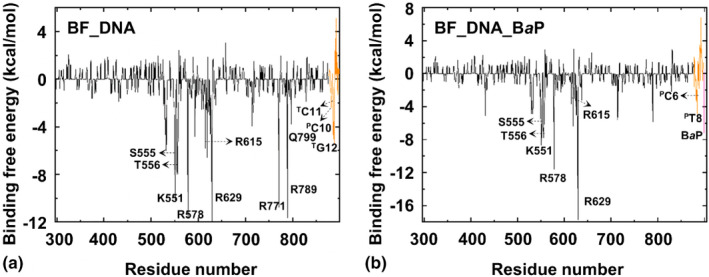
Energy decomposition and identification of key residues for the BF_DNA (a) and BF_DNA_BaP (b) systems. Black, orange, and pink are, respectively, used to represent proteins, nucleic acid sequences, and benzo[α]pyrene [BaP].

To explain how the interactions between Polymerase I and nucleic acid sequences weaken, Figure 7 shows the decomposition of their binding energies at residue and base levels. In the BF_DNA system, the recognition region is mainly distributed in Ser555‐Arg578, Arg615‐Arg629, and Arg771‐Gln799 of proteins, as well as ^P^C10, ^T^C11, and ^T^G12 of nucleic acids. While in the BF_DNA_BaP system, the strong binding effect of Arg771‐Gln799, ^T^G12, ^P^C10, and ^T^C11 disappeared, which was related to the strong spatial repulsion effect of BaP binding to this site.

#### 3.4.3 Decreasing the pore size of DNA replication terminal

**FIGURE 8:**
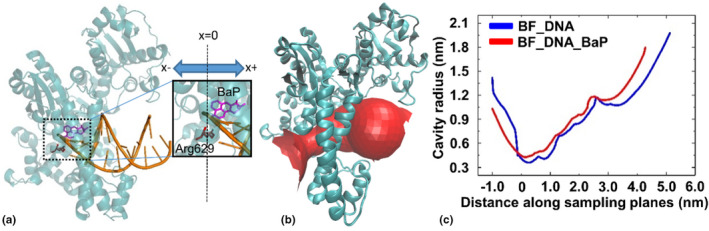
Comparative hole analyses of recognition interface between protein with nucleic acid in the BF_DNA and BF_DNA_BaP systems. (a) Three‐dimensional (3D) diagram of molecular interactions, the position of Arg629 is set to the origin of the *x*‐axis in panel C; (b) tunnel visualizations; and (c) cavity radius profiles vs. distance along sampling plane in the two systems.

Figure 8 shows the change of tunnel shape and cavity radius on the recognition surface between nucleic acid sequences and Polymerase I after BaP covalent binding. In the present BF_DNA and BF_DNA_BaP systems, the cavity starts from Arg629 inside the molecule and continues to extend outwards. It is defined here that when the cavity radius is greater than 2 nm, the tunnel is considered to have ended and entered the open solvent environment. As shown from Figure 8a, the BaP‐chelated DNA terminus—the starting point of DNA replication—is embedded near Arg629 of Polymerase I. Moreover, the insertion of BaP expands the binding pocket of the nucleic acid sequence (corresponding to the right side of the origin in Figure 8a,c), while its reaction and extension channel (corresponding to the left side of the origin) is compensatively shrunk, which is not conducive to the continuous replication of nucleic acid. By observing the first and second adjacent residues on tunnel surface, as expected, these key residues in the two systems were mainly located in Arg771‐GLN799, agreeing well with the result from energy decomposition in Figure 7. These residues play an important role in the recognition of protein with nucleic acid; especially, the movement of these rings directly affects the shape and cavity radius of nucleic acid binding channels.

In summary, a possible cytotoxicity mechanism of BaP is proposed. First of all, BaP covalently binds to ^T^G12 of double‐stranded nucleic acid to form DNA_BAP adduct, altering the conventional weak noncovalent interactions of double‐stranded nucleic acid chain, then disrupting the inherent stable molecular recognition between Polymerase I and nucleic acid sequences. In particular, after BaP binding, the ample space necessary for the movement of the extension ends is reduced, and the larger steric hindrance is not conducive to the DNA replication of Polymerase I.

## 4 CONCLUSION

The concentration database of BaP in Chinese food samples was first constructed; then, two models—point assessment and probability assessment—were used to evaluate its dietary exposure risk for college students in Bashu area; finally, the possible toxicity mechanism was revealed. Comparison of BaP concentration in different food samples shows that both edible oil and oil‐processed food pose a great threat to human health. The higher MOE values from the point assessment indicate that dietary exposure to BaP was generally safe; however, the results of the ILCR, a probabilistic assessment parameter, show that the average cancer risk is roughly between acceptable and severe. In terms of subdivided population, the proportion of medium‐risk and high‐risk of core and noncore district populations in Bashu area was 74.30/6.33% and 68.12/5.52%; the risk was slightly higher for female and those with graduate degrees than for male and those with bachelor degrees or below. A possible toxicity mechanism of BaP has been proposed through MD simulations: although BaP binding does not affect the global motion pattern of nucleic acid sequences, it significantly reduces the local weak noncovalent interactions; stable molecular recognition between Polymerase I and nucleic acid sequences is partially destroyed, especially by slightly changing the pore structure at the contact interface. Given that high‐temperature frying, smoking, and barbecuing are common cooking methods in southwest China, the emergence of BaP is inevitable. So, we put out four tips for dietary advice: (1) Take cooking strategies that reduce the production of BaP, such as shortening the frying time and lowering the oil temperature to 160°,180°; (2) improve the dietary habit, do not eat overfried food in a short time; (3) implement toxicity profiling of BaP for specific objects, especially for high‐risk groups, i.e., urban women with high degree; (4) proper combination of medicine and food homologous decoction contributing to relieve liver and kidney damage caused by an excessive intake of BaP. This study not only helps to guide the establishment of a reasonable and healthy diet, such as low intake of oil and oil‐processed food, but also proposes a possible carcinogenic mechanism of BaP possessing a certain theoretical significance.

## FUNDING INFORMATION

This work was supported by the National Key Research and Development Program of China (2018YFC1602101); the Project of Chongqing Key Laboratory of Environmental Materials and Restoration Technology (CEK1803); the Project of Sichuan Traditional Chinese Medicine Administration (2018KF006); Sichuan Science and Technology Program (2019YFH0054); and the Project of Chengdu Science and Technology Bureau (2016‐XT00‐00023‐GX).

## CONFLICT OF INTEREST

All the authors declare no conflict of interest, financial or otherwise.

## Supporting information


Table S1

Appendix S1

Appendix S2
