# Supplementary material for: Study on dietary intake, risk assessment, and molecular toxicity mechanism of benzo[α]pyrene in college students in China Bashu area
Source: Food Sci Nutr. 2022 Aug 9;10(12):4155–67. doi: 10.1002/fsn3.3007 (PMC9731532; doi:10.1002/fsn3.3007)
Supplement: Supplementary file 1 — Table S1 [file FSN3-10-4155-s002.docx]

Table S1 Occurrence of BaP in different foods in China

| **Food varieties** | **limit standard (μg·kg^-1^)** | **Sampling time (year)** | **Sampling province** |
| --- | --- | --- | --- |
| Rice | ≤5.0 | 2012, 2016, 2018 | Fujian, Ningxia, Hubei |
| Wheat | ≤5.0 | 2016, 2018 | Ningxia, Hubei |
| Fried meat products | ≤5.0 | 2009, 2017 | Fujian, Ningxia, Hubei |
| Roasted Pork | ≤5.0 | 2014, 2020 | Guangdong, Sichuan |
| Roasted mutton | ≤5.0 | 2009, 2020 | Chongqing, Sichuan |
| Roast beef | ≤5.0 | 2014, 2017 | Guangdong, Guangxi |
| Broiler chicken | ≤5.0 | 2014, 2017, 2020 | Guangdong, Guangxi, Sichuan |
| Grilled seafood | ≤5.0 | 2009, 2017, 2020 | Chongqing, Guangxi, Sichuan |
| Bacon and sausage | ≤5.0 | 2013-2015, 2020 | Hunan, Guangdong, Sichuan |
| Peanut oil | ≤10.0 | 2015, 2017, 2019, 2020 | Nei Mongol, Hubei, Shaanxi, Gansu |
| Olive oil | ≤10.0 | 2015, 2017 | Nei Mongol, Hubei |
| Sesame oil | ≤10.0 | 2013-2016, 2020 | Henan, Shanxi, Nei Mongol, Ningxia, Gansu |
| Rapeseed oil | ≤10.0 | 2015-2017, 2020 | Hubei, Hunan, Ningxia, Guizhou, Gansu |
| Blend oil | ≤10.0 | 2015, 2017 | Nei Mongol, Hubei |
| Soybean oil | ≤10.0 | 2009, 2011, 2014-2017, 2020 | Heilongjiang, Jilin, Shanxi, Nei Mongol, Ningxia, Hubei, Gansu |
| Corn oil | ≤10.0 | 2015, 2017, 2020 | Nei Mongol, Hubei, Gansu |
| Sunflower oil | ≤10.0 | 2015-2017 | Nei Mongol, Ningxia, Hubei |
